# A Practical Numerical Approach to Characterizing Non-Linear Shrinkage and Optimizing Dimensional Deviation of Injection-Molded Small Module Plastic Gears

**DOI:** 10.3390/polym13132092

**Published:** 2021-06-25

**Authors:** Xiansong He, Wangqing Wu

**Affiliations:** 1 State Key Laboratory of High Performance Complex Manufacturing, Central South University, Lushan South Road 932, Changsha 410083, China; csuxshe@csu.edu.cn; 2 School of Mechanical and Electrical Engineering, Central South University, Lushan South Road 932, Changsha 410083, China

**Keywords:** small module plastic gears, non-linear shrinkage, injection molding, dimensional deviation

## Abstract

This paper was aimed at finding out the solution to the problem of insufficient dimensional accuracy caused by non-linear shrinkage deformation during injection molding of small module plastic gears. A practical numerical approach was proposed to characterize the non-linear shrinkage and optimize the dimensional deviation of the small module plastic gears. Specifically, Moldflow analysis was applied to visually simulate the shrinkage process of small module plastic gears during injection molding. A 3D shrinkage gear model was obtained and exported to compare with the designed gear model. After analyzing the non-linear shrinkage characteristics, the dimensional deviation of the addendum circle diameter and root circle diameter was investigated by orthogonal experiments. In the end, a high-speed cooling concept for the mold plate and the gear cavity was proposed to optimize the dimensional deviation. It was confirmed that the cooling rate is the most influential factor on the non-linear shrinkage of the injection-molded small module plastic gears. The dimensional deviation of the addendum circle diameter and the root circle diameter can be reduced by 22.79% and 22.99% with the proposed high-speed cooling concept, respectively.

## 1. Introduction

In recent decades, with the development of polymer manufacturing technology [1,2,3,4], the share of plastic gears in the gear market has gradually increased. Compared with metal gears, plastic gears have obvious advantages [5] such as low weight, low cost, self-lubrication, strong ability to absorb shock and vibration, etc. Therefore, it is widely used in frontier such as medical devices, aerospace, and industrial robots [6,7]. The latest additive manufacturing and 3D printing processes manufacturing is a great breakthrough for plastic gears manufacturing, but it is mainly used in small batch production [8]. At present, plastic gears are mainly manufactured by injection molding [9,10,11] because of its low production cost and short production cycle. The injection molding technology for conventional gears is mature and can basically meet the use requirements. However, for micro-nano manufacturing grade small module plastic gears (gears with module less than or equal to 1 mm are usually called small module gears), there are still many challenges to be addressed in micro-injection molding [12,13]. The biggest issue faced by small module plastic gears is the serious shortage of dimensional accuracy. Due to the inherent shrinkage of polymer [14,15,16], plastic parts could experience a non-linear shrinkage in the stage of pressure holding and cooling, resulting in deviation between the final plastic part and the mold cavity geometry. Moreover, the overall size of small module plastic gears is smaller, and the shape deviation has a more significant impact on its dimensional accuracy. Low dimensional accuracy will affect the stability of gear transmission, produce vibration, and noise, and accelerate the fracture of tooth root and gear wear [17,18]. It will inevitably shorten the service life of small module plastic gears [19].

The key to solve the problem of insufficient dimensional accuracy of small module plastic gears is to control the non-linear shrinkage of plastic parts in the molding process. Different from conventional module gears, small module gears have smaller specific surface area and smaller convection and radiation heat dissipation resistance during cooling. The heat transfer at the interface [20,21,22,23] during melt molding is complex and changeable, so the uneven heat transfer at various parts of the gear is more obvious. The premise of shrinkage control is to understand the shrinkage characteristics of small module plastic gears. However, because the forming process of plastic gears is not visualized and the experimental conditions are difficult to control, it is difficult for researchers to quantitatively describe the shrinkage characteristics of plastic gears in the production process. It is also impossible to selectively control the influencing factors in the molding process of small module plastic gears. To study the forming process of small module plastic gears visually, it is of great significance to use computer numerical simulation technology [24,25,26] to simulate the forming shrinkage behavior of gears. It can not only reduce the cost of research and development experiments but also greatly shorten the production cycle. At present, the finite element analysis software Moldflow can simulate the polymer manufacturing process completely and accurately [27,28].

Shrinkage behavior of plastic parts is related to many factors such as processing equipment, material properties, mold structure, plastic parts geometry, and injection molding process parameters [29,30,31]. For gears whose dimensions have been determined in practical production, the key concern is the influence of injection molding process parameters on shrinkage. Gear injection molding process parameters are mainly divided into three factors: time, pressure, and temperature. Various factors affecting the cooling shrinkage process of small module plastic gears should be comprehensively considered through characteristics analysis [32,33]. At the same time, there are many injection molding process parameters, which will consume a lot of time during test adjustment. Many scholars have studied the process parameters of conventional gear injection molding and found that Taguchi method plays a great role in reducing the number of tests and adjusting the process parameters [34,35,36,37]. Moreover, it is found that many parameters have little influence on the warpage and shrinkage deformation of microstructure parts, which is helpful to further reduce the complexity of research. 

In view of the challenges mentioned above, Zhu et al. found that combination of shrinkage directions and shrinkage distances of points on an injection-molded part determine shrinkage ratios for various dimensions of the part, and shrinkage directions are more influential to shrinkage ratios of dimensions, which offered a unique approach to understand the shrinkage principles of injection-molded parts [38]. Ghazali et al. successfully analyzed the plastic gear injection molding process using MPI software [39]. Ramkumar et al. found that Taguchi optimization and ANOVA method are very useful in determining the most important molding process parameters that affect volume shrinkage and optimizing control parameters to achieve minimum part shrinkage [40]. Eghbal Hakimian et al. also used the Taguchi method to assist numerical simulation to analyze the influence of injection molding parameters and thermoplastic composites on the maximum warpage of injection-molded micro gears [41]. However, the injection gate of the micro gear is set in the gear teeth, which is inconsistent with the actual production. Jain et al. developed a 3D model of industrial size plastic gear using pro E and analyzed the flow of plastic gears [42]. Mehat et al. used MPI simulation software to simulate and analyze the injection molding process of polymer gear. Using the Taguchi optimization method, it was found that the process parameters that have great influence on gear shrinkage are melt temperature, packing pressure, and packing time [43].

At present, most researchers’ research on shrinkage of plastic gears mainly stays at the stage of large and medium module. There is little research on the non-linear shrinkage characteristics of small module plastic gears. Furthermore, the research target parameters only stay in the stage of maximum warping shrinkage deformation of gears, and there is no targeted research on dimensional deviation. Therefore, it is a great challenge to optimize the molding accuracy of small module plastic gears at present.

In this paper, aiming at the non-linear shrinkage problem of injection-molded small modulus plastic gear, a practical numerical approach was proposed to characterize the nonlinear shrinkage and optimize dimensional deviation of small module plastic gears. Moldflow was used to simulate the warping shrinkage deformation and intuitively predict the gear-forming shrinkage process. The 3D shrinkage model of gear was successfully exported, and the shrinkage characteristics of small module plastic gears were studied. Based on the characteristics research, taking the key dimensional deviation of small module gears (addendum circle diameter and root circle diameter) as the response target, the orthogonal experiments were designed. The influence relationship between key process parameters and key dimensional deviation of small module gears and its influence contribution ratio was obtained. Under the guidance of the best combination of parameters in orthogonal experiments, a high-speed cooling technology of moldboard-cavity combination for small module plastic gears was proposed. Although there is no experimental verification in this paper, the viscosity model and constitutive model adopted by the material are all from Moldflow material database. Based on the wide application of Moldflow in practical engineering and compared with the research results of other scholars, the obtained results provide credible theoretical basis for die cavity design and shrinkage control and have great guiding value.

## 2. Materials and Methods

### 2.1. Material

**Figure 1:**
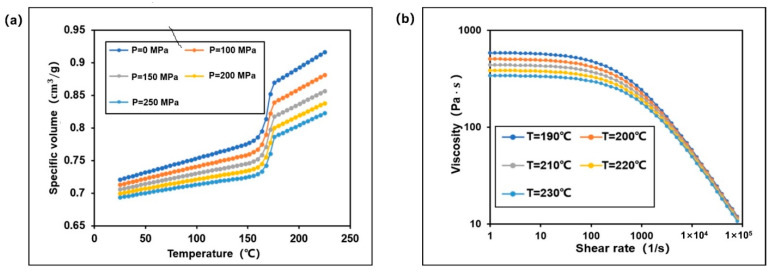
(**a)** The PVT curve (**b**) Curve of shear rate and viscosity.

The properties of polymer materials are directly related to the final molding quality of small module plastic gears. The selection of plastic gear materials should comprehensively consider three aspects: service performance, process performance and economic cost [44]. Polyformaldenyde (POM) [45,46], as the most used thermoplastic engineering plastic for gear products, has excellent comprehensive properties. In this study, POM Delrin 500 P produced by DuPont was selected as the research material of small module plastic gears in Moldflow material library, which is the most used polymer material for small module gears. The physical properties of POM and Cross-William-Landel-Ferry (Cross-WLF) viscosity model are shown in Table 1 and Table 2, respectively. In addition, the Cross-WLF viscosity model represent the correlation of temperature, shear rate, and pressure based on the viscosity of thermoplastic materials. Figure 1a shows the (Pressure, Volume, Temperature) PVT variation curve of the material, and Figure 1b shows the viscosity curve of the material in molten state. Specifically, the PVT variation curve describes the temperature and pressure relationship of the polymer in the whole processing range. Meanwhile, the curve of shear rate and viscosity describes about the viscosity change in the flow process of polymer, which is also supplemented in Table 2.

### 2.2. Gear 3D Modeling

**Figure 2:**
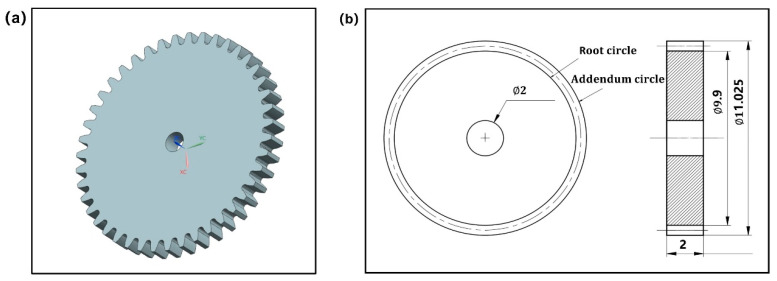
(**a**) The 3D model of the designed gear. (**b**) Geometry and specification of the designed gear: module = 0.25 mm, pressure angle = 20, number of teeth = 20, face width = 10 mm, and modification coefficient = 0.45.

In this study, to meet the requirement of finite element simulation analysis accuracy of small module plastic gears, a commercial-computer aided design software Unigraphics (UG) with more powerful performance was selected to build the 3D model of the designed gear [47]. In order to more directly and clearly analyze the non-linear shrinkage characteristics of gear and avoid the influence of gear type, the most common involute spur gear was selected as the research object. The gear 3D model built in this study was a high precision small module gear. The 3D model and geometric parameters of the designed gear are shown in Figure 2.

### 2.3. Numerical Analysis

**Figure 3:**
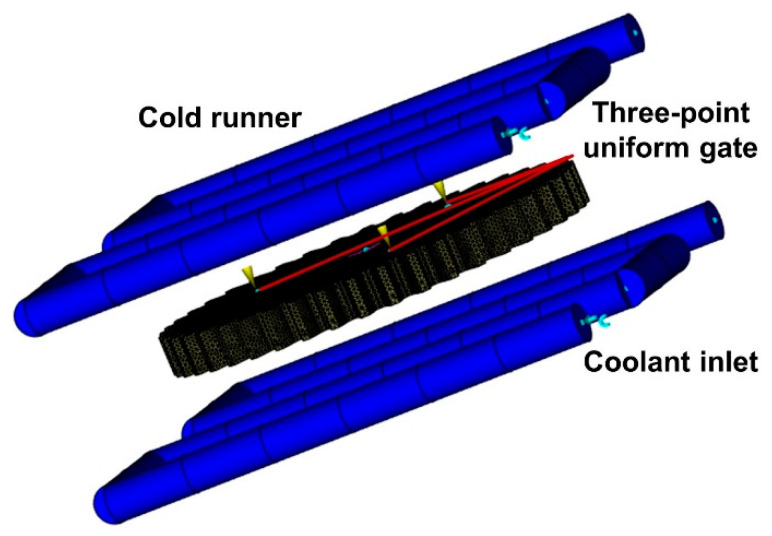
Gear mold flow analysis simulation model.

In this study, the gear numerical model established by Zhu and other researchers is combined and improved [38,39,40,41,42,43]. The proposed numerical approach was realized in a commercial finite element analysis code under the brand name of Moldflow. First, the 3D model of designed gear established in UG was imported into Moldflow in STP format. Then, according to the designed gear size, appropriate grid density was set for double-layer grid division. The grid statistics are shown in Table 3. The grid accuracy was enough to simulate the gear model. To study the non-linear shrinkage of small module plastic gears, the analysis sequence of “cooling filling and pressure packing warpage” was selected. The gear had a central hole structure and a central symmetrical geometric shape. For full fill and even cool, the most common three-pin uniform gate was designed. In addition, the gear was cooled by four sets of straight-through cooling circuits of upper and lower templates, which can be produced by 3D printing. The simulation model of the designed gear is shown in Figure 3. First, the characteristics analysis pre-test research of small module plastic gears was carried out under the default process parameter level shown in Table 4. 

### 2.4. Orthogonal Experiments

Since the cavity geometry, polymer materials, and injection molding machine models are not easy to change, the main way to control the shrinkage of small module gears is to control key process parameters. It is very important to study the influence of relationship between injection molding process parameters and molding accuracy of small module plastic gears. There are many process parameters involved in the injection molding process of small modulus plastic gear, so it is not suitable to use single factor analysis. Orthogonal experimental analysis method should be adopted for this research [48]. Orthogonal experiment is another design method to study multi-factors and multi-levels, which selects some representative points from the comprehensive experiment according to orthogonality. Using orthogonal experiment, not only the number of experiments is greatly reduced but also the calculation of statistical analysis is simplified. 

Therefore, to better study the influence of process parameters on gear shrinkage, the research focuses on the controllable process parameters that affect filling, pressure keeping, and cooling process. In this study, the key parameters such as melt temperature, mold temperature, packing pressure, packing time, and coolant temperature were selected for orthogonal experimental design. The selection level of each parameter was determined according to the recommended polymer process parameter level in the material library, as shown in Table 5. Moreover, the $L_{25}\left(5^{5}\right)$ orthogonal array, as shown in Table 6, was selected to simulate the cooling filling and pressure packing warpage according to the simulation model in Figure 3.

### 2.5. Range Analysis and ANOVA

The range analysis can visually assess the influence level of each process parameter on the key dimensional deviation of the gear. In addition, the optimal levels can be obtained. The range (*R*) equation was calculated according to the following equation.

(1) $$\begin{array}{c} R_{j}=max\left(\bar{K_{j1}},\bar{K_{j2}},\cdots ,\bar{K_{jm}}\right)-min\left(\bar{K_{j1}},\bar{K_{j2}},\cdots ,\bar{K_{jm}}\right) \end{array}$$

where $\bar{K_{jm}}$ is the mean sum of experiment indexes, *j* is the column number, and *m* is the level number.

To evaluate the specific contribution percentages (*C%*) of each molding process parameter to the key dimensional deviation of small module plastic gears, the analysis of variance (ANOVA) of gear shrinkage results was carried out by Minitab 18 software. The *C%* was calculated according to the following equation.

(2) $$C_{i}\%=\frac{SS_{i}}{SST}\times 100$$

where $C_{i}$ is the percentage contribution (*C%*) of each factor, $SS_{i}$ is the sum of the square of each factor, i is the factor number (where i = 1 represents factor A and where i = 5 represents the factor E), and *SST* is the sum of the squares of the effects (treatments), i.e., $SST=\sum SS_{i}$. The percentage contribution of each factor represents the influence rate.

## 3. Results

### 3.1. Shrinkage Characteristics Analysis of Small Module Plastic Gears

**Figure 4:**
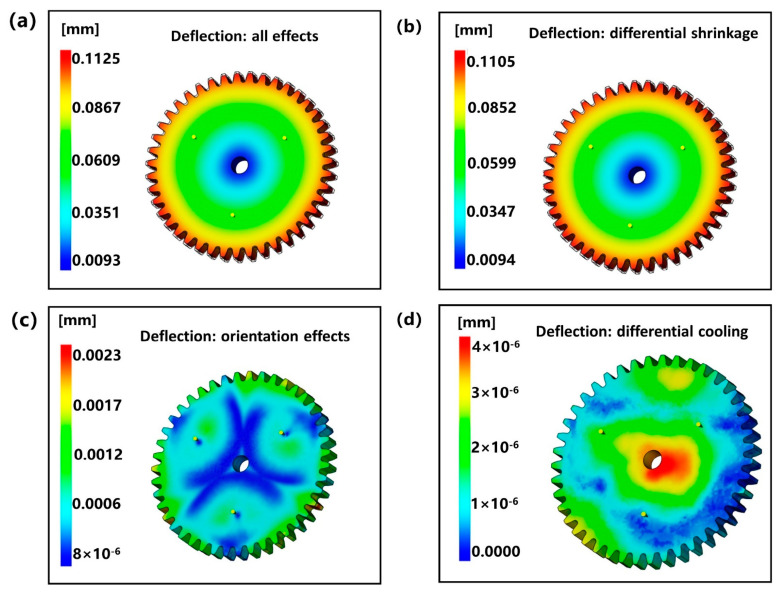
The shrinkage nephogram of the designed gear: (**a**) total deformation, (**b**) deformation caused by uneven shrinkage, (**c**) deformation caused by orientation effect, and (**d**) deformation caused by uneven cooling.

The warpage deformation results of the designed gear were obtained by Moldflow software simulation analysis. Warpage deformation is the direct cause of gear dimensional deviation. Warpage deformation is mainly caused by three effects, which are uneven shrinkage, uneven cooling, and orientation effect. Figure 4 is the nephogram of the whole warpage deformation effect of the designed gear, and Figure 4a is the total deformation of the gear, with the maximum deformation of 0.1125 mm. Figure 4b shows the deformation of gears caused by uneven shrinkage. It can be clearly seen that the deformation caused by uneven shrinkage is very close to the overall deformation of the gear, and the maximum deformation is 0.1105 mm. Figure 4c shows the deformation of gears caused by orientation effect. It can be seen that the cloud image of orientation effect presents three-point central symmetry distribution, which is closely related to the distribution of gates, and the maximum deformation is 0.0023 mm. Figure 4d shows the deformation of the gear caused by uneven cooling, and the maximum deformation is only 4 × 10^−6^ mm, which can be ignored basically. It is concluded that the deformation of small module plastic gear mainly depends on uneven shrinkage. It explains theoretically that the results of warping and shrinkage in Erfan Oliaei et al. are very close and have the same changing trend [37]. Moreover, the effects of uneven cooling and orientation have little influence on the warpage deformation of the gear, which can be ignored directly. Therefore, it is necessary to analyze its non-linear shrinkage characteristics to study the dimensional accuracy of small module plastic gears.

**Figure 5:**
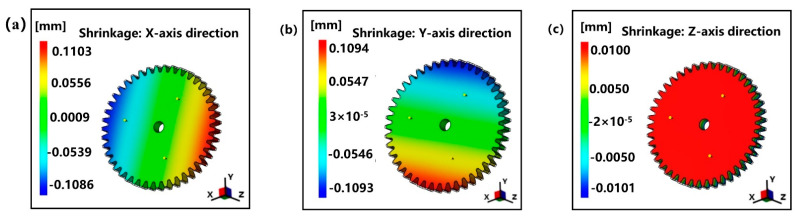
The shrinkage nephogram of the designed gear in different directions: (**a**) shrinkage in X-axis direction, (**b**) shrinkage in Y-axis direction, and (**c**) shrinkage in Z-axis direction.

Next, the shrinkage of small module plastic gears was divided into three directions: X, Y, and Z, as shown in Figure 5. With the structural center of the gear as the origin, Figure 5a,b shows the shrinkage of the designed gear in the X-axis direction and the Y-axis direction. It shows that the shrinkage trends of the two directions are very close, and the shrinkage of the gear in the X-axis direction and Y-axis direction at both ends of the origin presents symmetrical distribution. Figure 5c shows the shrinkage of the gear in the Z-axis direction, i.e., the thickness direction of the gear. It shows that the maximum shrinkage is only 0.01 mm. For small module plastic gears, the shrinkage in the thickness direction is very small, so the research can focus on the radial shrinkage of gears. In addition, the shrinkage of the gear is symmetrical about the center of the structure. To simplify the calculation, some areas can be selected along the radius direction as the research object to replace the overall shrinkage.

**Figure 6:**
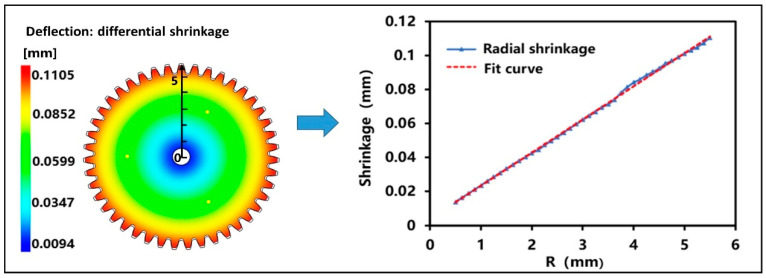
The radial shrinkage of the designed gear.

During the forming process, the polymer melt follows the isotropic and centripetal shrinkage law centered on the gate. However, the small modulus plastic gear adopts three-point uniform distribution injection mode, and the gate is not in the center of the whole structure. As shown in Figure 6, it shows that the shrinkage of the designed gear still presents roughly isotropic shrinkage, and the shrinkage situation is the same at the same diameter. To study the radial shrinkage of small module plastic gears, the radius of the gear was divided equally on the scale of 0.20 mm, and the shrinkage at each point was measured. The results shown in Figure 6 were obtained. It is concluded that the radial shrinkage of gears presents a linear relationship and follows the law of isotropic centripetal shrinkage under the three-point uniform distribution injection mode. 

**Figure 7:**
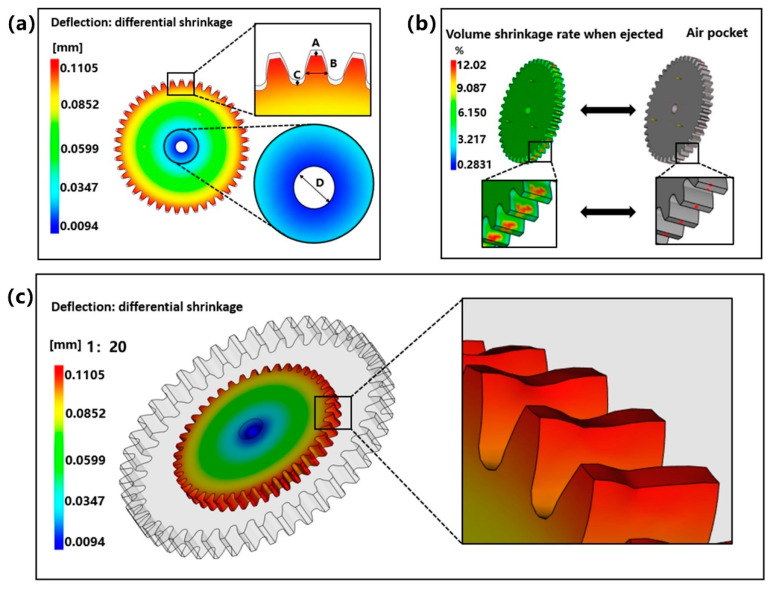
(**a**) The radial shrinkage nephogram of the designed gear. (**b**) Comparison of the volume shrinkage rate of the gear ejection and air-pocket analysis. (**c**) Gear shrinking scale 1:20 enlargement processing.

To further understand the shrinkage characteristics of small module plastic gears, the analysis object can be specific to the key positions of the gear. Figure 7a shows the radial shrinkage nephogram of the designed gear. First, the designed gear with the shrinkage model was compare. It shows that the shrinkage of gear teeth is the largest. By enlarging partial teeth and the central hole, it can be clearly seen that the shrinkage at tooth top A and tooth root C is very large, while the shrinkage at B near the pitch circle is very small. Moreover, there is almost no shrinkage at the center hole. It is concluded that the shrinkage of gear is mainly concentrated in addendum circle and root circle. Therefore, when we study the shrinkage of small modulus plastic gears, we should focus on the shrinkage of addendum circle and root circle. Figure 7b compares the volume shrinkage when the gear is ejected with the air-pocket and shows that the volume shrinkage in the center of the gear tooth thickness direction is larger. In addition, it shows that there are bubbles in this part. Therefore, it is known that the existence of bubbles has a certain effect on the shrinkage deformation of gears. Because the gear teeth have relatively large shrinkage and relatively small structure, the gear shrinkage is expanded according to the ratio of 1:20, and it enlarges the partial teeth, as shown in Figure 7c. It shows that the radial shrinkage of the center part of the gear teeth along the thickness direction is obviously larger than that of both sides and the tooth profile angle changes accordingly. This phenomenon of wasting has a great influence on the tooth profile accuracy of small module plastic gears.

**Figure 8:**
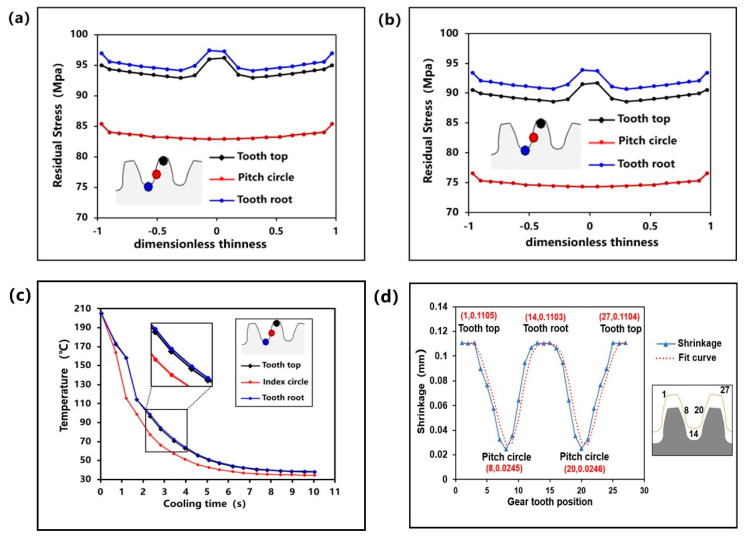
(**a**) The residual stress in the first main direction. (**b**) The residual stress in the second main direction. (**c**) Temperature curve of cooling process of the key positions. (**d**) The edge shrinkage in a gear tooth cycle.

Due to various external factors, the disorder and relaxation of molecular chains in melt materials are destroyed. It makes the melt in an unstable state and produces residual stress [49,50]. In the process of molecular chain relaxation or recrystallization, the stress will be released, which leads to the shrinkage of related positions. Figure 8a,b shows the stress in the first main direction (the residual stress in the orientation direction before ejection) and in the second main direction (the residual stress in the vertical direction of the first main direction before ejection) of the key positions, respectively, when the gear was ejected. Moreover, the x-axis label “dimensionless thickness” is a value for measuring the thickness of a part, which ranges from -1 to 1, where 0 represents the center position in the thickness direction, and -1 and 1 refer to both sides. It is known that the residual stress at the addendum circle and the root circle is larger than that at the pitch circle. In addition, the residual stress in the center of tooth thickness direction is greater than that in other positions. This also explains the phenomenon that the addendum circle and root circle of small module plastic gears shrink greatly, but the pitch circle shrinks little, and the phenomenon of waisting. Figure 8c is the temperature curve of the cooling process of the designed gear. It is known that the cooling rate of the addendum circle and root circle is equivalent to that of the pitch circle. Therefore, the cooling time is longer before the gear geometry is stabilized. To a certain extent, it was confirmed that cooling rate is the most influential factor on the non-linear shrinkage of the injection-molded small module plastic gears. The faster the cooling rate, the shorter the cooling time and the smaller the shrinkage. It is also consistent with the research results of Chil-Chyuan Kuo et al. [51]. Figure 8d shows a graph of edge shrinkage in a gear tooth cycle. The edge of gear teeth was divided into several segments evenly and the shrinkage corresponding to each point was measured. It shows that the contraction relationship of gear teeth presents a gear tooth function relationship.

### 3.2. Analysis of Orthogonal Experiments Results

**Figure 9:**
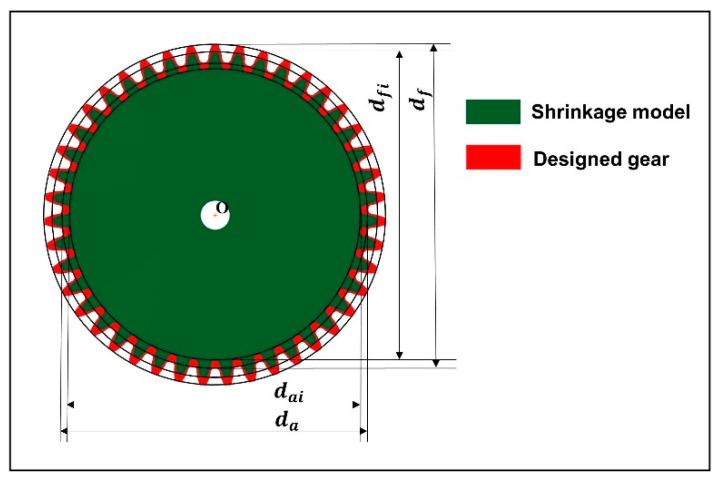
Schematic diagram of measurement method.

**Figure 10:**
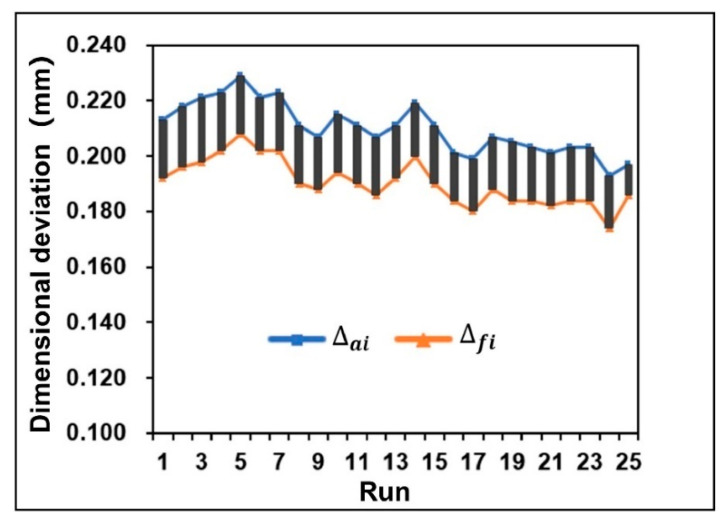
Comparison of dimensional deviation results of orthogonal experiments.

After orthogonal experiments, the 3D shrinkage model of the designed gear was exported in a 1:1 ratio in STP format. Through characteristics analysis, it is known that the biggest shrinkage parts of small module plastic gears are the addendum circle and the root circle, and the shrinkage presents isotropic centripetal shrinkage. To quantificationally characterize the shrinkage and forming dimensional accuracy of gears, the dimensional deviation of the addendum circle diameter and root circle diameter were taken as the response targets. The key dimensions of 3D shrinkage model of the designed gear were measured by analyzing geometric attribute module in UG. The schematic diagram of measurement method is shown in Figure 9. The dimensional deviation of addendum circle diameter and root circle diameter was calculated by Equations (3) and (4), respectively. The results are shown in Table 7.

(3) $$\Delta_{ai}(i=1,2,3\ldots \ldots 25)=d_{a}-d_{ai}$$

(4) $$\Delta_{fi}(i=1,2,3\ldots \ldots 25)=d_{f}-d_{fi}$$

where $d_{a}$ is the diameter of addendum circle, $d_{f}$ is the diameter of root circle, and i is the number of tests. Figure 10 shows the results of the difference of key dimensional deviation of each group. It is obvious that the dimensional deviation of gear varies significantly under different process parameters. By comparing the dimensional deviation of addendum circle diameter with that of root circle diameter, it can be found that they have the same variation trend. Therefore, it was confirmed that the diameter of the addendum circle and the diameter of the root circle contract synchronously, which also reduces the difficulty of overall adjustment and control of gear shrinkage to a certain extent.

Then, the results of orthogonal experiments were visually analyzed by range analysis. The results are shown in Table 8. According to the dimensional deviation of addendum circle diameter, the influence degree from top to bottom is melt temperature, coolant temperature, packing time, packing pressure, and mold temperature. According to the dimensional deviation of root circle diameter, the influence degree from high to low is melt temperature, coolant temperature, packing time, mold temperature, and packing pressure. The optimal levels combination of process parameters is $A_{5}\;B_{4}\;C_{4}\;D_{1}\;E_{1}$ (melt temperature 230 °C, mold temperature 100 °C, packing pressure 70 MPa, packing time 15 s, and coolant temperature 10 °C) under the condition of minimum deviation of diameter of addendum circle and diameter of root circle.

**Figure 11:**
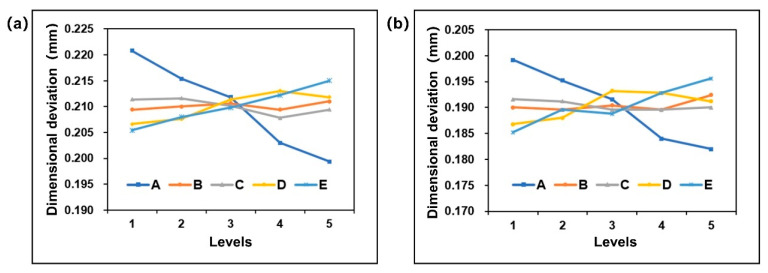
Plots of process parameters effects: (**a**) the dimensional deviation of the addendum circle and (**b**) the dimensional deviation of the root circle.

To describe the influence law of injection molding process parameters on key dimensional deviation more intuitively, the horizontal axis of process parameters was taken as the abaxial axis, and the key dimensional deviation was taken as the vertical axis. The response relationship between the change of process parameters and the diameter deviation of addendum circle diameter and root circle diameter was obtained, as shown in Figure 11a,b, respectively. Since there is a synchronous variation relationship between the diameter of addendum circle and the diameter of root circle, they show roughly the same rule. The results show that there is a negative correlation between the size deviation of addendum circle diameter and root circle diameter and melt temperature and there is a positive correlation between them and coolant temperature.

The ANOVA results are shown in Table 9. As for the dimensional deviation of the addendum circle, the melt temperature has the greatest influence, reaching 75.1938%; followed by the cooling liquid temperature and the packing time, 13.3236% and 7.6066%, respectively; and the mold temperature and the packing pressure have the smallest contribution percentages, which are 0.4845% and 2.3740%, respectively. As for the dimensional deviation of the root circle, the melt temperature has the greatest contribution percentage, reaching 66.0272%; followed by the coolant temperature and the packing time, 19.4926% and 10.2104%, respectively; and the mold temperature and packing pressure have very little contribution percentage. It is also consistent with the result that Mehat et al. found that melt temperature is the most important process parameter for gear shrinkage [42]. Based on the above analysis, it is known that melt temperature has the greatest influence on the key dimensional deviation of gears, followed by coolant temperature and packing time, while mold temperature and packing pressure have very little influence.

### 3.3. Optimization of Dimensional Deviation

**Figure 12:**
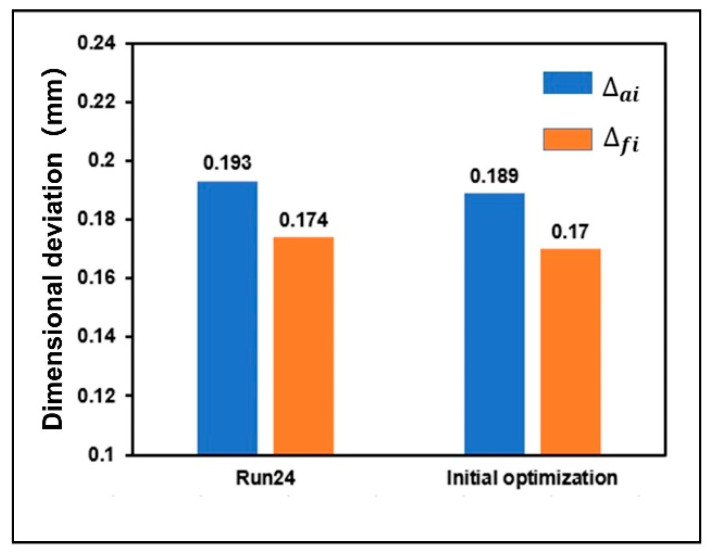
Comparison results of initial optimization of gear dimensional deviation.

According to the results of orthogonal experiments analysis, the optimal parameter combination of the designed gear is $A_{5}\;B_{4}\;C_{4}\;D_{1}\;E_{1}$. It was set as the forming process parameters of gear initial optimization simulation model. After obtaining the initial optimized shrinkage model of the designed gear, the dimensional deviation was compared with the minimum dimensional deviation of orthogonal experiments, as shown in Figure 12. The dimensional deviation of the addendum circle and the root circle decreased by 2.072% and 2.353%, respectively. This also verifies the accuracy of orthogonal experiments to some extent.

**Figure 13:**
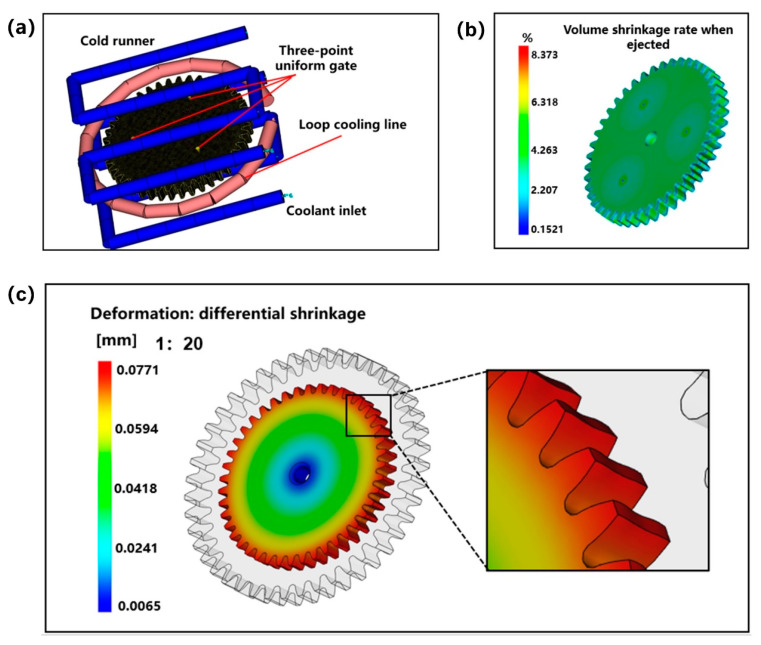
(**a**) Gear high-speed cooling simulation model. (**b**) High-speed cooling: volume shrinkage rate when the gear is out. (**c**) High-speed cooling: gear shrinking scale 1:20 enlargement processing.

Through shrinkage characteristics analysis, it was found that the shrinkage of gear teeth is the largest, and as the cooling rate increases, the cooling time decreases and the shrinkage decreases. Fangcheng Xiao et al. [52] found that cooling system is a key influencing factor of molding shrinkage rate. The molding shrinkage rate can be adjusted by optimizing the mold’s cooling system. Therefore, under the optimal parameter combination, the cavity loop cooling pipeline was added. A plate-cavity combined high-speed cooling model, as shown in Figure 13a, was designed to accelerate the cooling of gear teeth. Further, the volume shrinkage of the gear during ejection is shown in Figure 13b. The results show that the shrinkage rate of the gear during ejection is greatly reduced, the shrinkage of each part is more even, and there is no large volume shrinkage in the center of the tooth thickness direction. Then, the shrinkage of the small module plastic gear is enlarged according to the ratio of 1:20, and the gear teeth are locally enlarged, as shown in Figure 13c. It shows that the radial shrinkage of gear teeth in the thickness direction is relatively even, and there is no big sudden change, thus avoiding the occurrence of waist shrinkage.

**Figure 14:**
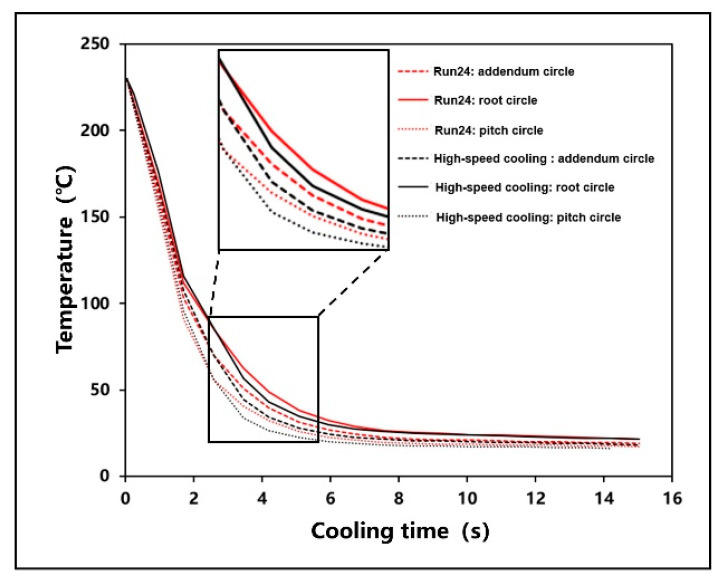
Comparison of temperature curves of high-speed cooling technology cooling process.

**Figure 15:**
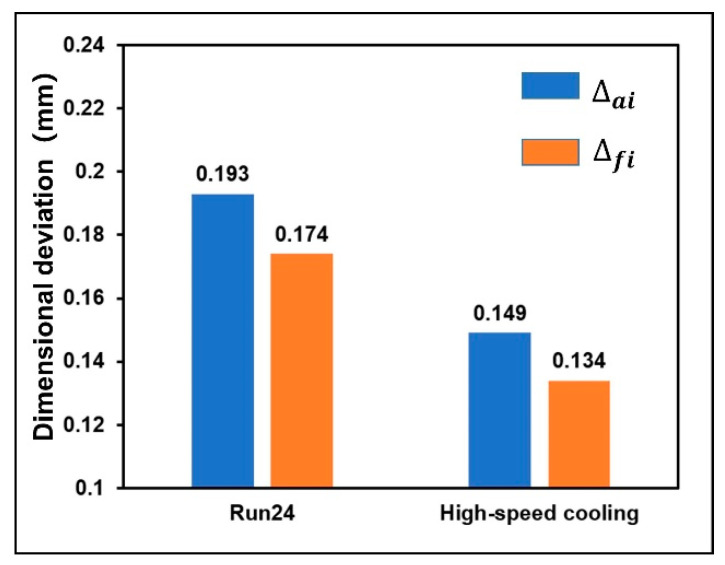
Comparison of optimization results of high-speed cooling technology.

Figure 14 shows the temperature change of key positions during cooling process under the Run 24 test and high-speed cooling technology. It was found that the cooling rate of addendum circle, root circle, and pitch circle increase, and the cooling time is greatly reduced under high-speed cooling technology. Finally, the dimensional deviation of addendum circle diameter and root circle diameter were measured and calculated, as shown in Figure 15. The results show that the dimensional deviation of the addendum circle is 0.149 mm, which is reduced by 22.79%, the dimensional deviation of the root circle is 0.134 mm, which is reduced by 22.99%.

## 4. Conclusions

Non-linear shrinkage in the forming process of small module plastic gears leads to serious shortage of dimensional accuracy. In this study, a practical numerical approach was proposed to characterize the non-linear shrinkage and optimize the dimensional deviation of the small module plastic gears. First, based on Moldflow simulation technology, the shrinkage characteristics of injection-molded small module plastic gears were analyzed. Then, the influence relationship between molding process parameters and key dimensional deviation were obtained through analysis of orthogonal experiments. Finally, the key dimensional deviation was optimized by the high-speed cooling technology of gears plate-cavity combination. Key findings of this study include:

1. The dimensional accuracy of small module plastic gears mainly depends on the non-linear shrinkage in the forming process. The shrinkage of gear teeth is the largest, especially the diameter of addendum circle and root circle.

2. The volume shrinkage rate in the center of the tooth thickness direction of small module plastic gear is too large locally, which leads to waist shrinkage. Moreover, it was found that the residual stress in the top circle and root circle of gear teeth is obviously larger than that in the pitch circle, and a larger residual stress appears in the center of gear teeth in the thickness direction. 

3. The 3D shrinkage model of small module plastic gears was exported. It was confirmed that cooling rate is the most influential factor on the non-linear shrinkage of the injection-molded small module plastic gears. The faster the cooling rate, the shorter the cooling time and the smaller the shrinkage.

4. Melt temperature has the greatest influence on the key dimensional deviation of small module plastic gears. In addition, the optimal levels combination of process parameters is $A_{5\;}B_{4\;}C_{4\;}D_{1\;}E_{1}$ (melt temperature 230 °C, mold temperature 100 °C, packing pressure 70 MPa, packing time 15 s, and coolant temperature 10 °C) under the condition of minimum deviation of diameter of addendum circle and diameter of root circle. 

5. The high-speed cooling technology of gears plate-cavity combination was proposed. Under the high-speed cooling of gear teeth, it was found that the dimensional deviation of the addendum circle and the root circle are reduced by 22.79% and 22.99%, respectively, and the shrinkage of each part is more even. At the same time, the waist shrinkage disappeared.

In general, the influence of forming process parameters on the critical dimension deviation of gears was studied, and the shrinkage of small module plastic gears is well predicted. At the same time, the high-speed cooling technology of small modulus plastic gear template-cavity combination was proposed, which makes the key dimensional deviation of gear reduced obviously. Therefore, this approach provides a theoretical basis for die cavity design and shrinkage control in practical production of small module plastic gears.

## Figures and Tables

**Table 1:** Polyformaldenyde (POM) physical properties table.

Factor	Value
Melt density (g/cm^3^)	1.1027
Solid density (g/cm^3^)	1.387
Hardness (HRO)	92
Thermal conductivity (W/m·K)	0.29
Water absorption (%)	1.4
Coefficient of friction	0.25

**Table 2:** The Cross-William-Landel-Ferry (WLF) viscosity model.

Factor	Value
n	0.1958
Tau∗(Pa)	378,000
D1 (Pas)	7.29 × 10^11^
D2 (K)	223.15
D3 (K/Pa)	0
A1	25.44
A2~(K)	51.6

**Table 3:** Grid statistics.

Factor	Value
Number of elements	51,322
Minimum aspect ratio	1.54
Maximum aspect ratio	8.39
Average aspect ratio	1.16
Match percentage (%)	90.9
Reciprocal percentage (%)	92.7

**Table 4:** Pre-experiment process parameters.

Melt Temperature (°C)	Mold Temperature (°C)	Packing Pressure (MPa)	Packing Time (s)	Coolant Temperature (°C)
210	80	60	25	25

**Table 5:** The level of injection molding process parameters.

Factor	Parameter	Level 1	Level 2	Level 3	Level 4	Level 5
A	Melt temperature (°C)	190	200	210	220	230
B	Mold temperature (°C)	70	80	90	100	110
C	Packing pressure (MPa)	40	50	60	70	80
D	Packing time (s)	15	20	25	30	35
E	Coolant temperature (°C)	10	15	20	25	30

**Table 6:** $L_{25}\left(5^{5}\right)$ orthogonal array.

Run#	Factor				
	A	B	C	D	E
1	1	1	1	1	1
2	1	2	2	2	2
3	1	3	3	3	3
4	1	4	4	4	4
5	1	5	5	5	5
6	2	1	2	3	4
7	2	2	3	4	5
8	2	3	4	5	1
9	2	4	5	1	2
10	2	5	1	2	3
11	3	1	3	5	2
12	3	2	4	1	3
13	3	3	5	2	4
14	3	4	1	3	5
15	3	5	2	4	1
16	4	1	4	2	5
17	4	2	5	3	1
18	4	3	1	4	2
19	4	4	2	5	3
20	4	5	3	1	4
21	5	1	5	4	3
22	5	2	1	5	4
23	5	3	2	1	5
24	5	4	3	2	1
25	5	5	4	3	2

**Table 7:** Results of orthogonal experiment.

Run#	*d_ai_* (mm)	*d_fi_* (mm)	Δ*_ai_* (mm)	Δ*_fi_* (mm)
**1**	10.812	9.708	0.213	0.192
**2**	10.807	9.704	0.218	0.196
**3**	10.804	9.702	0.221	0.198
**4**	10.802	9.698	0.223	0.202
**5**	10.796	9.692	0.229	0.208
**6**	10.804	9.698	0.221	0.202
**7**	10.802	9.698	0.223	0.202
**8**	10.814	9.710	0.211	0.190
**9**	10.818	9.712	0.207	0.188
**10**	10.810	9.706	0.215	0.194
**11**	10.814	9.710	0.211	0.190
**12**	10.818	9.714	0.207	0.186
**13**	10.814	9.708	0.211	0.192
**14**	10.806	9.700	0.219	0.200
**15**	10.814	9.710	0.211	0.190
**16**	10.824	9.716	0.201	0.184
**17**	10.826	9.720	0.199	0.180
**18**	10.818	9.712	0.207	0.188
**19**	10.820	9.716	0.205	0.184
**20**	10.822	9.716	0.203	0.184
**21**	10.824	9.718	0.201	0.182
**22**	10.822	9.716	0.203	0.184
**23**	10.822	9.716	0.203	0.184
**24**	10.832	9.726	0.193	0.174
**25**	10.828	9.714	0.197	0.186

**Table 8:** The range analysis.

Index Level	Δ*_ai_*					Δ*_fi_*				
	A	B	C	D	E	A	B	C	D	E
$\bar{K_{j1}}$	0.2208	0.2094	0.2114	0.2066	0.2054	0.1992	0.1900	0.1916	0.1868	0.1852
$\bar{K_{j2}}$	0.2154	0.2100	0.2116	0.2076	0.2080	0.1952	0.1896	0.1912	0.1880	0.1896
$\bar{K_{j3}}$	0.2118	0.2106	0.2102	0.2114	0.2098	0.1916	0.1904	0.1896	0.1932	0.1888
$\bar{K_{j4}}$	0.2030	0.2094	0.2078	0.2130	0.2122	0.1840	0.1896	0.1896	0.1928	0.1928
$\bar{K_{j5}}$	0.1994	0.2110	0.2094	0.2118	0.2150	0.1820	0.1924	0.1900	0.1912	0.1956
$R_{j}$	0.0214	0.0016	0.0038	0.0064	0.0096	0.0172	0.0028	0.0020	0.0064	0.0104
**Optimal level**	5	1 and 4	4	1	1	5	2 and 4	3 and 4	1	1

**Table 9:** The ANOVA results.

Factor	Δ*_ai_*					Δ*_fi_*				
	$SS_{i}$	DOF	MS	F	C%	$SS_{i}$	DOF	MS	F	C%
**A**	0.001552	4	0.000388	75.186	75.1938	0.001067	4	0.000267	44.467	66.0272
**B**	0.000010	4	0.000003	0.496	0.4845	0.000027	4	0.000007	1.133	1.6708
**C**	0.000049	4	0.000012	2.357	2.3740	0.000018	4	0.000004	0.733	1.1139
**D**	0.000157	4	0.000039	7.628	7.6066	0.000165	4	0.000041	6.867	10.2104
**E**	0.000275	4	0.000069	13.326	13.3236	0.000315	4	0.000079	13.133	19.4926
**Error**	0.000021	4	0.000005	75.186	1.0174	0.000024	4	0.000006	44.467	1.4851
**Total**	0.002064	24				0.001616	24			

## Data Availability

Not applicable.
